# 基于TCGA数据库的*EGFR*突变型与野生型肺腺癌患者免疫微环境的差异性分析

**DOI:** 10.3779/j.issn.1009-3419.2021.102.15

**Published:** 2021-04-20

**Authors:** 光胜 朱, 永文 李, 睿峰 施, 松林 徐, 子禾 张, 培俊 曹, 琛 陈, 红雨 刘, 军 陈

**Affiliations:** 1 300052 天津，天津医科大学总医院肺部肿瘤外科 Department of Lung Cancer Surgery, Tianjin Medical University General Hospital, Tianjin 300052, China; 2 300052 天津，天津市肺癌研究所，天津市肺癌转移与肿瘤微环境重点实验室 Tianjin Key Laboratory of Lung Cancer Metastasis and Tumor Microenvironment, Tianjin Lung Cancer Institute, Tianjin Medical University General Hospital, Tianjin 300052, China

**Keywords:** 肺肿瘤, 表皮生长因子受体, 免疫治疗, 免疫浸润, 免疫微环境, Lung neoplasms, Epidermal growth factor receptor, Immunotherapy, Immune infiltration, Immune microenvironment

## Abstract

**背景与目的:**

肺癌是一种具有高发病率与高死亡率的恶性肿瘤，腺癌是其中一个重要的组织亚型。表皮生长因子受体（epidermal growth factor receptor, *EGFR*）突变是肺腺癌患者重要的驱动基因。EGFR-酪氨酸激酶抑制剂（tyrosine kinase inhibitor, TKI）对*EGFR*敏感突变的患者疗效显著。而免疫治疗作为新兴的治疗却不能使*EGFR*突变患者获益，其中的机制研究尚不明确，并集中于EGFR与程序性死亡受体-配体1（programmed cell death-ligand 1, PD-L1）表达之上，而我们推测与两类患者不同的免疫微环境有关。

**方法:**

从癌症基因组图谱（The Cancer Genome Atlas, TCGA）数据库收集肺腺癌数据集，下载临床信息资料及基因表达谱资料。通过TIMER2.0计算TCGA数据库中免疫相关淋巴细胞浸润情况。并对*EGFR*突变型与野生型患者进行基因集富集分析。

**结果:**

临床特征分析显示*EGFR*突变更频繁发生于女性以及未吸烟患者中。免疫浸润分析显示*EGFR*突变患者通常具有更高的肿瘤相关成纤维细胞、普通髓系祖细胞、造血干细胞、效应CD4^+^ T细胞、自然杀伤T细胞浸润；具有更低的记忆B细胞、初始B细胞、浆细胞、浆细胞样树突状细胞、记忆CD4^+^ T细胞、CD4^+^辅助性T细胞2、CD8^+^ T细胞、中心记忆CD8^+^ T细胞、初始CD8^+^ T细胞浸润。我们发现CD8^+^ T细胞、自然杀伤T细胞、记忆B细胞和造血干细胞在肿瘤中浸润的程度越高则患者预后越好（*Log-rank*检验，*P*=0.017、0.009, 3、0.018和0.016）。同时CD4^+^辅助性T细胞2在肿瘤中浸润的程度越高则患者预后越差（*Log-rank*检验，*P*=0.016）。基因集富集分析的结果显示，相比较EGFR野生型肺腺癌患者而言，*EGFR*突变患者的自然杀伤细胞介导的对肿瘤细胞的免疫应答的正调控、自然杀伤细胞激活参与免疫反应、在自然杀伤细胞介导的对肿瘤细胞的免疫应答这三条与自然杀伤细胞有关的通路上均处于下调状态，而参与免疫应答的细胞因子分泌的正调节这条通路为上调。

**结论:**

*EGFR*突变患者肿瘤微环境缺乏有效的杀伤肿瘤的效应细胞并出现了效应细胞功能失调。这可能是*EGFR*突变患者免疫治疗疗效差的潜在原因。

## Abstract (en)

**Background and objective:**

Lung cancer is a malignant with high incidence and mortality and adenocarcinoma is among the most popular subtypes. Epidermal growth factor receptor (*EGFR*) mutation is one of the most important driver mutations for lung adenocarcinoma and EGFR-tyrosine kinase inhibitor (TKI) will benefit those patients with sensitive *EGFR* mutations. Recently, immune checkpoint inhibitor (ICI) therapy, provide a new breakthrough treatment for lung cancer patients. Whereas immunotherapy as an emerging treatment does not benefit patients with *EGFR* mutations, for which mechanistic studies are poorly defined and focused on the link of *EGFR* mutations and programmed cell death-ligand 1 (PD-L1) expression, we speculate that the different immune microenvironment associated with the two classes of patients.

**Methods:**

Lung adenocarcinoma datasets were collected from the Cancer Genome Atlas (TCGA) database, and clinical information and gene expression profiles were downloaded. The immune related lymphocyte infiltration in TCGA database were generated through timer 2.0 GSEA was used to analyze the difference of pathway expression between *EGFR* mutant patients and wild type patients.

**Results:**

*EGFR* mutation was more frequently among women and never smokers. Immunoinfiltration analysis showed that patients with *EGFR* mutation tends to have more tumor associated fibroblasts, common myeloid progenitor cells, hematopoietic stem cells, effector CD4^+^ T cells and natural killer T cells infiltration, and less memory B cells, naïve B cells, plasma B cells, plasmacytoid dendritic cells, memory CD4^+^ T cells, CD4^+^ helper T cells 2, naive CD8^+^ T cells, CD8^+^ T cells and central memory CD8^+^ T cells infiltration. Moreover, patients with more infiltration of CD8^+^ T cells, natural killer T cells, memory B cells and hematopoietic stem cells, tends have better prognosis (*Log-rank* test, *P*=0.017, 0.0093, 0.018, 0.016). However, the patients with more CD4^+^ T th2 infiltration in the tumor tends to have worse prognosis (*Log-rank* test, *P*=0.016). Furthermore, the results of gene set enrichment analysis showed that compared with the lung adenocarcinoma patients with *EGFR* wild type, the three pathways positive regulation of natural killer (NK) cell-mediated immune response to tumor cells, NK cell activation involved in immune response, and NK cell-mediated immune response to tumor cells related to natural killer cells in patients with *EGFR* mutation were down regulated, while the pathway the positive regulation of cytokine secretion involved in immune response was up-regulated in *EGFR* mutation patients.

**Conclusion:**

The tumour microenvironment of patients with *EGFR* mutations lacks potent tumour killing effector cells and appears dysfunctional with effector cells. This may be a potential reason for the poor efficacy of immunotherapy in patients with *EGFR* mutations.

肺癌是世界上最常见的恶性肿瘤之一，也是全球癌症相关死亡的最常见原因，每年有超过一百万人死于肺癌^[1]^。根据组织学类型不同，肺癌分为两个主要亚型：非小细胞肺癌（non-small cell lung cancer, NSCLC）和小细胞肺癌（small cell lung cancer, SCLC）分别占所有病例的85%和15%^[2]^。肺腺癌是最常见的NSCLC，近年来，表皮生长因子受体酪氨酸激酶抑制剂（epidermal growth factor receptor tyrosine kinase inhibitors, EGFR-TKIs），显著改善*EGFR*阳性突变的肺腺癌患者的无进展生存期（progression-free survival, PFS）和总生存期（overall survival, OS）^[3]^。

免疫检查点抑制剂（immune checkpoint inhibitor, ICI）的使用是近年来肺癌治疗的又一项重大突破。已有的数据表明，在程序性死亡配体1（programmed cell death ligand 1, PD-L1）表达阳性的肺癌患者中，ICI单药或联合化疗的疗效，超过了化疗药。因此，依据PD-L1表达水平的不同，ICI单药或ICI联合化疗已经成为肺癌患者的一线治疗选择方案。另一方面，在具有*EGFR*突变的肺腺癌患者中，ICIs的治疗效果普遍不佳。比如最近的一项临床研究探索了*EGFR*突变的肺癌患者一线使用ICIs的效果。这项II期单臂试验对PD-L1表达≥1%的*EGFR*突变NSCLC患者进行每3周静脉注射200 mg派姆单抗（Pembrolizumab）治疗，在11例患者接受治疗后，由于缺乏疗效而该研究提前结束，客观缓解率（objective response rate, ORR）为0%，结果令人十分失望^[4]^，而产生如此结果的机制尚不明确，推测与两类患者不同的免疫微环境有关。因此，最新的美国国立综合癌症网络（National Comprehensive Cancer Network, NCCN）临床指南及中国抗癌协会临床肿瘤学协作中心（Chinese Society of Clinical Oncology, CSCO）临床指南均指出，*EGFR*突变阳性肺腺癌患者首选的治疗方案是EGFR-TKI；对驱动基因突变阴性、PD-1表达阳性患者，ICI单药或ICI联合化疗可以成为一线治疗方案。

癌症基因组图谱（The Cancer Genome Atlas, TCGA）作为目前最大的癌症基因信息数据库，包含了临床样本数据以及多组学的数据，其中包含了包括基因表达数据、拷贝数变异、DNA甲基化、单核苷酸多态性（single nucleotide polymorphism, SNP）的数据。

本研究拟通过TCGA公共数据集，在分析研究*EGFR*突变肺腺癌患者与野生型肺腺癌患者的临床特征差异的基础上，进一步分析其免疫微环境水平的差异，初步探讨*EGFR*突变肺腺癌患者与野生型肺腺癌患者在接受ICIs治疗时产生的不同治疗效果的可能的发生机制，为未来可能的治疗方案提供线索和思路。

## 1 材料与方法

### 1.1 数据资料收集

从TCGA数据库官网（https://portal.gdc.cancer.gov/）下载566例肺腺癌患者的临床资料、单核苷酸改变的数据以及RNA-seq的信息。通过Cbioportal在线数据库（http://www.cbioportal.org/）获得了肺腺癌肿瘤突变分布图（图 1）。

**图 1:**
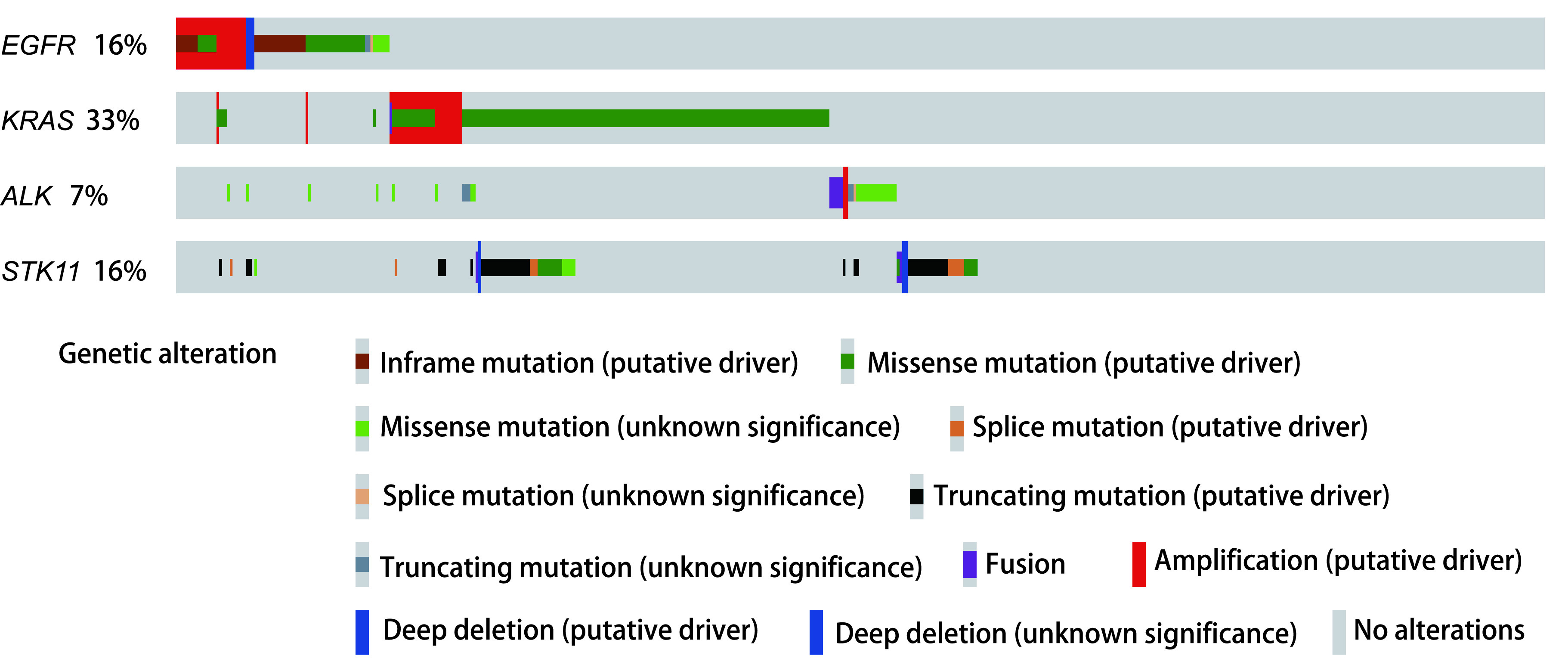
肺腺癌患者基因突变分布图 Oncoplot of lung adenocarcinoma

### 1.2 数据集筛选

根据单核苷酸改变的数据，利用样本中是否出现*EGFR*突变的信息进行筛选，仅保留501例TCGA数据集中包含*EGFR*突变的信息和对应的RNA-seq的病例。并将其通过EGFR是否突变分为*EGFR*突变组与野生型组。

### 1.3 免疫相关淋巴细胞浸润计算

通过TIMER2.0（http://timer.comp-genomics.org/）获得通过四种方法（CIBERSORT, CIBERSORT ABS, QUANTISEQ, XCELL）^[5]^计算得出的TCGA数据库中免疫相关淋巴细胞浸润结果。

### 1.4 功能富集分析

利用R语言“clusterProfiler”程序包对野生型组和*EGFR*突变组进行基因集富集分析（Gene Set Enrichment Analysis, GSEA）。

### 1.5 统计学分析

使用SPSS 21.0软件进行统计学分析。临床信息相关性分析，组间比较采用卡方检验及*Fisher*确切概率法，采用乘积极限法（*Kaplan-Meier*）绘制生存曲线、对数秩检验（*Log-rank*）比较不同样本的生存曲线。*P* < 0.05为差异有统计学意义。

## 2 结果

### 2.1 EGFR野生型和突变型肺腺癌患者的相关临床资料分析

本研究从TCGA数据集下载、整理、分析了501例肺腺癌患者的临床数据和其对应的信息，其中，男性232例（46%），女性269例（54%），中位年龄65岁；存在吸烟史的407例（81%），无吸烟史的67例（13%），27例患者的吸烟史未被提及；*EGFR*突变型67例（13%），EGFR野生型434例（87%）。如表 1所示，*EGFR*突变型和野生型肺腺癌患者在年龄、肿瘤大小、有无淋巴结转移、有无远处转移、临床分期方面均无统计学差异，而*EGFR*突变型较野生型肺腺癌更频繁发生于女性患者（突变型女性：n=48，72%；野生型女性：n=221，51%）（*P*=0.003）；*EGFR*突变型较野生型肺腺癌更频繁发生于从未吸烟患者（突变型从未吸烟患者：n=24，36%；野生型从未吸烟患者：n=43，10%）（*P* < 0.001）。进一步采用*Kaplan-Meier* Plotter方法分析*EGFR*突变型与野生型肺腺癌患者的生存差异，如图 2所示，二者在生存时间上未见显著的统计学差异（*Log-rank*检验，*P*=0.22，图 2）。

**表 1:** EGFR野生型和突变型肺腺癌患者的相关临床资料分析 Analysis of clinical data of patients with *EGFR*-mutation and EGFR-wild lung adenocarcinoma

Characteristic		*EGFR*		*P*
		Mutation (*n*=67)	Wild (*n*=434)	
Gender	Male	19 (28%)	213 (49%)	0.003
	Female	48 (72%)	221 (51%)	
Age (yr)	< 60	18 (27%)	116 (27%)	0.942
	≥60	46 (67%)	303 (70%)	
Smoking status	Ever	39 (58%)	368 (85%)	< 0.001
	Never	24 (36%)	43 (10%)	
T stage	T1/T2	57 (85%)	380 (88%)	0.505
	T3/T4	10 (15%)	52 (12%)	
N stage	N0	36 (54%)	285 (66%)	0.069
	N1/N2/N3	29 (43%)	141 (32%)	
M stage	M0	45 (67%)	292 (67%)	0.847
	M1	3 (4%)	22 (5%)	
Stage	Ⅰ/Ⅱ	48 (72%)	343 (79%)	0.151
	Ⅲ/Ⅳ	19 (28%)	89 (21%)	
Missing values exsit in The Cancer Genome Atlas. The percentages of the mutant group in the table are all calculated with 67 as the denominator; the percentages of the wild group in the table are all calculated with 434 as the denominator. EGFR: epidermal growth factor receptor.				

**图 2:**
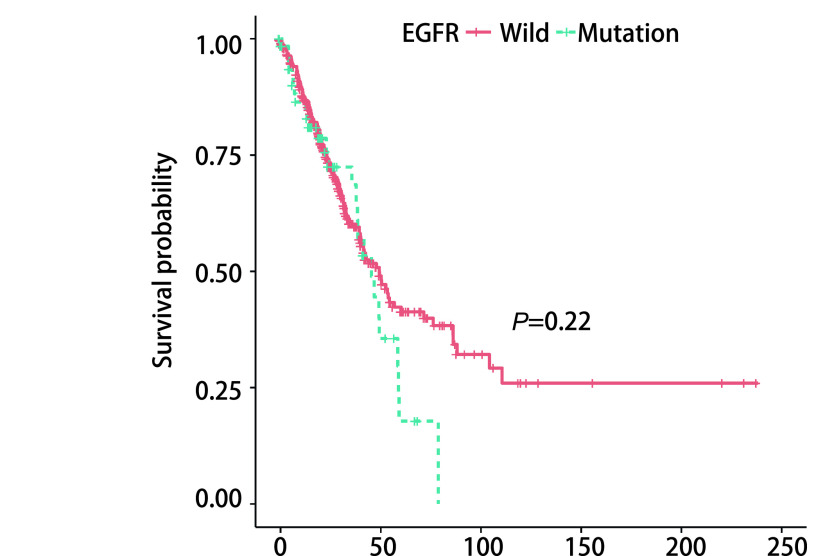
*EGFR*是否突变与肺腺癌患者预后的关系 The relationship between *EGFR* mutation and prognosis of patients with lung adenocarcinoma

### 2.2 EGFR野生型组和*EGFR*突变组患者肺癌组织免疫相关细胞浸润水平分析

本研究通过XCELL法计算获得样本的免疫相关淋巴浸润情况，使用R语言的“ggplot2”程序包分析了在*EGFR*突变型与野生型肺腺癌患者之中，免疫相关淋巴浸润情况的差异。我们发现*EGFR*突变患者趋于具有更高的肿瘤相关成纤维细胞、普通髓系祖细胞、造血干细胞、效应CD4^+^ T细胞、自然杀伤T细胞浸润；更低的记忆B细胞、初始B细胞、浆细胞、浆细胞样树突状细胞、记忆CD4^+^ T细胞、CD4^+^辅助性T细胞2、CD8^+^ T细胞、中心记忆CD8^+^ T细胞、初始CD8^+^ T细胞浸润（图 3A）。我们对67例*EGFR*突变患者的样本中细胞浸润情况做了相关性分析，发现除造血干细胞（*P* < 0.05）、肿瘤相关成纤维细胞（*P* < 0.05）、内皮细胞之间互相具有较强的相关性（*P* < 0.05），各免疫细胞浸润之间相关性不大（图 3B）。另外，已经有研究证明较低的CD8^+^ T细胞与较差的免疫治疗的疗效是相关的^[6]^。同时，CD8^+^ T细胞是ICIs治疗的直接效应细胞，CD8^+^ T细胞浸润高低将直接影响ICIs治疗对肿瘤细胞所带来的细胞毒性效应的高低。因此，本研究采用其他三种计算免疫浸润的方法验证了CD8^+^ T细胞浸润在*EGFR*突变型与野生型之间的差异，证实了*EGFR*突变型患者具有较低的CD8^+^ T细胞浸润（图 4）。结果显示，这可能是*EGFR*突变患者免疫治疗疗效差的因素之一。与此同时通过肿瘤突变分布图，我们发现EGFR野生型患者中*KRAS*基因突变比例较高（图 1）。

**图 3:**
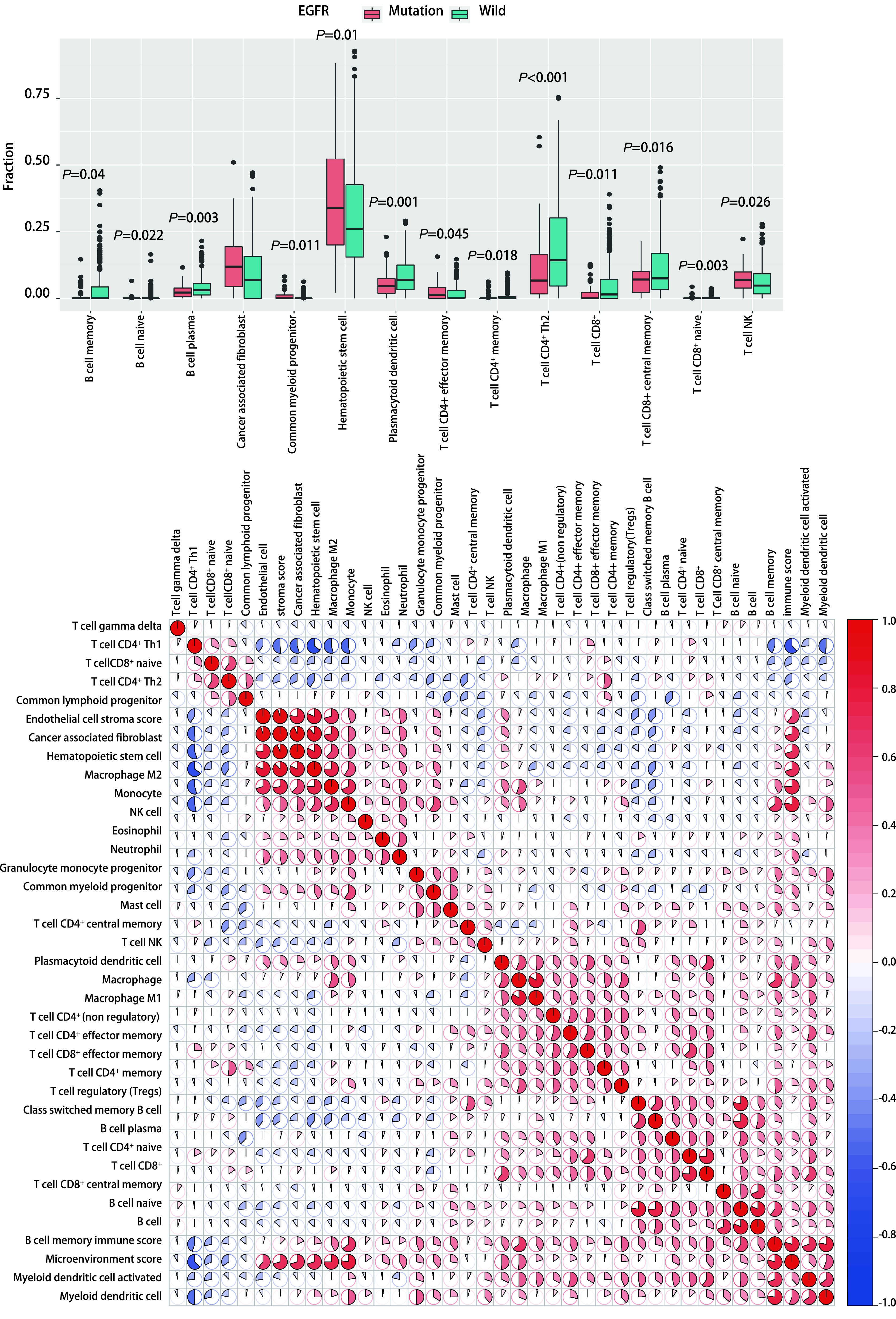
*EGFR*突变肺腺癌患者免疫微环境改变。A: 野生型组和*EGFR*突变组免疫相关细胞浸润水平的差异；B：对67例*EGFR*突变型肺腺癌患者免疫相关细胞浸润程度的相关性分析。 Changes of immune microenvironment in lung adenocarcinoma patients with *EGFR* mutation. A: differences in the infiltration levels of immune related cells between the wild-type group and the *EGFR* mutation group; B: Correlation analysis of the degree of immune related cell infiltration in 67 patients with *EGFR* mutant lung adenocarcinoma.

**图 4:**
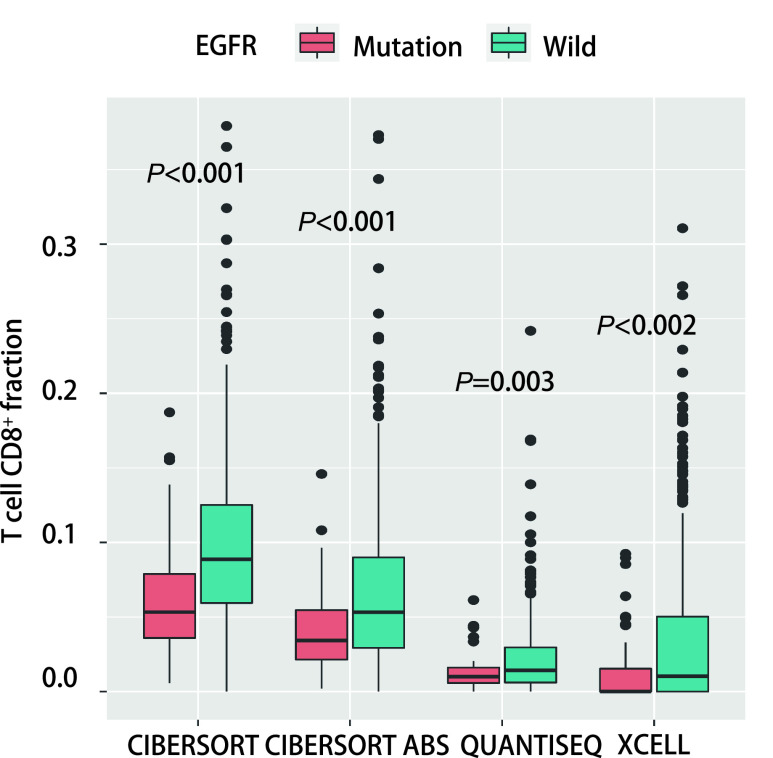
使用四种方法评估野生型组和*EGFR*突变组CD8^+^ T细胞浸润程度 Four methods were used to assess the degree of CD8^+^ T-cell infiltration in the wild-type group and the *EGFR* mutant group

### 2.3 免疫细胞浸润程度与生存预后分析

为了探索免疫细胞浸润程度与肺腺癌患者预后的关系，我们采用*Kaplan-Meier* Plotter方法分析各种在*EGFR*突变型与野生型浸润存在差异的免疫相关细胞浸润高低与生存预后的关系。我们发现在肺腺癌患者中，CD8^+^ T细胞、自然杀伤T细胞、记忆B细胞和造血干细胞在肿瘤中浸润的程度越高则患者预后越好（*Log-rank*检验，*P*=0.017、0.009, 3、0.018和0.016）（图 5A、5B、5D、5E）。而CD4^+^辅助性T细胞2在肿瘤中浸润的程度越高则患者预后越差（*Log-rank*检验，*P*=0.016）（图 5C）。这与往期的研究结果是类似的^[7]^，CD8^+^ T细胞、自然杀伤T细胞等有助于肿瘤免疫反应的细胞浸润增多与患者较好的预后密切相关，而CD4^+^辅助性T细胞2这种免疫抑制细胞对患者预后不利。

**图 5:**
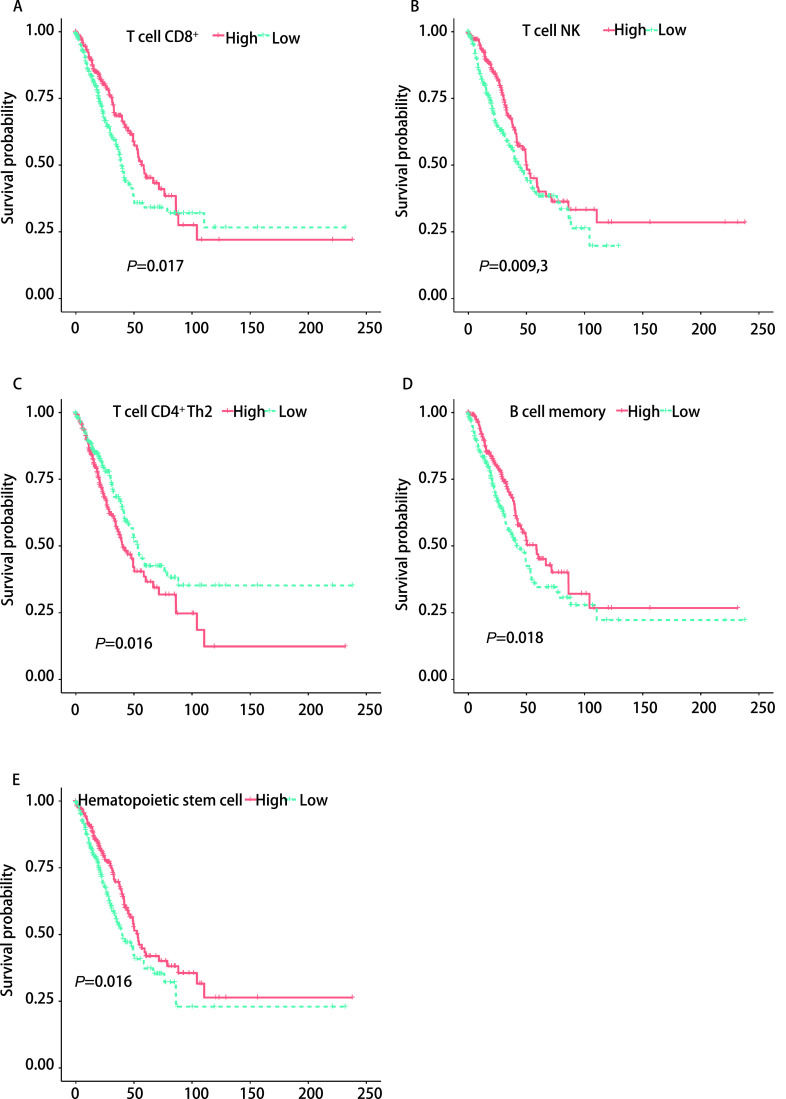
*EGFR*突变型与野生型浸润程度存在差异的免疫相关细胞与肺腺癌患者预后的关系 Immune related cells differentially infiltrating *EGFR* mutant versus wild type correlate with prognosis in lung adenocarcinoma patients

### 2.4 *EGFR*突变型和野生型肺腺癌患者基因集富集通路分析

为了分析*EGFR*突变型和野生型肺腺癌患者在免疫相关通路上的改变，本研究采用R语言“clusterProfiler”程序包对野生型组和*EGFR*突变组进行基因集富集分析。结果显示，*EGFR*突变组相对于野生型组，在免疫相关的通路上除了参与免疫应答的细胞因子分泌的正调节（positive regulation of cytokine secretion involved in immune response）通路中的关键基因为上调外，在自然杀伤细胞介导的对肿瘤细胞的免疫应答的正调控（positive regulation of natural killer cell mediated immune response to tumor cell）、自然杀伤细胞激活参与免疫反应（natural killer cell activation involved in immune response）、在自然杀伤细胞介导的对肿瘤细胞的免疫应答（natural killer cell mediated immune response to tumor cell）这三条与自然杀伤细胞有关的通路上的关键基因均处于下调状态（图 6）。

**图 6:**
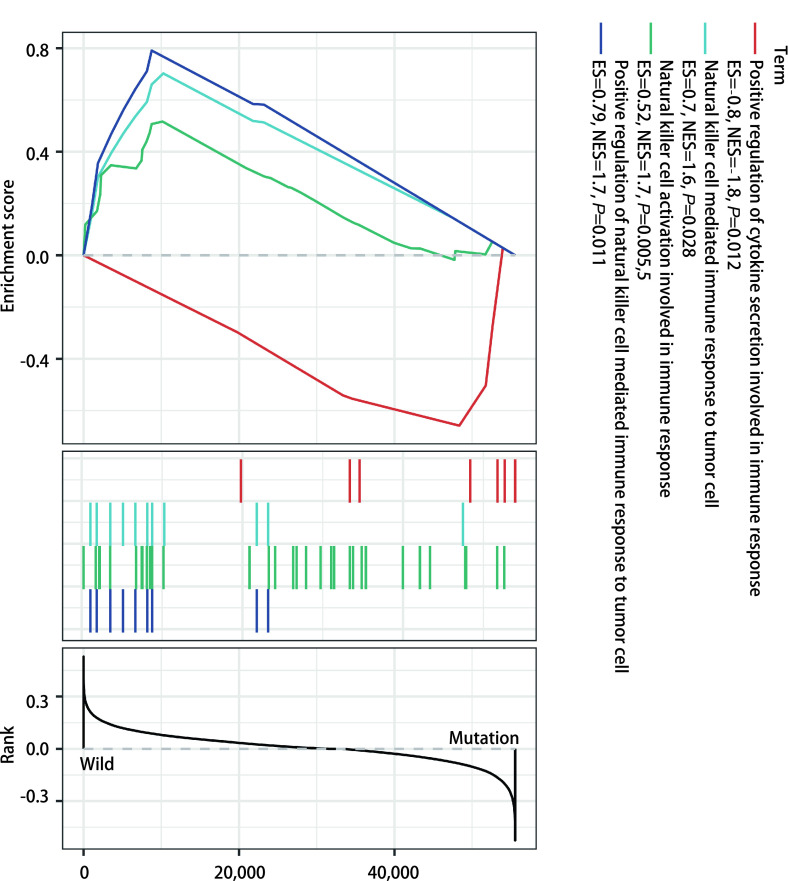
*EGFR*突变型和野生型肺腺癌患者基因集富集通路分析 Gene set enrichment pathway analysis of *EGFR* mutant and wild type lung adenocarcinoma patients

## 3 讨论

肺癌是中国发病率和死亡率最高的恶性肿瘤^[8]^，其中NSCLC占85%^[2]^。在NSCLC中，肺腺癌占据了很大的比重。对于存在*EGFR*敏感突变的晚期肺腺癌患者来说，临床指南建议EGFR-TKIs作为一线用药^[9-11]^。而ICIs已经展现出能使NSCLC患者长期获益的能力，并具有较轻的副作用的特点。临床前研究表明，EGFR激活可上调肿瘤细胞PD-L1的表达，从而诱导T细胞凋亡，促进*EGFR*突变NSCLC的免疫逃逸。然而，一些临床研究已经证明PD-1抑制剂治疗*EGFR*突变的NSCLC患者的疗效可能很差^[12, 13]^。本研究主要探讨了*EGFR*突变型与野生型患者肿瘤免疫浸润的改变，以探讨*EGFR*突变型患者接受ICIs时效果差的潜在机制。

本研究发现，与野生型肺腺癌患者相比，*EGFR*突变型患者的肿瘤的CD8^+^ T细胞浸润程度较低。这可能与EGFR野生型患者中*KRAS*基因突变比例较高有关（图 3）。而较低的CD8^+^ T细胞与较差的免疫治疗的疗效是相关的^[6]^。同时我们也发现较低的CD8^+^ T细胞浸润与肿瘤患者更差的预后是存在联系的（图 5A）。并且，我们使用了四种方法计算CD8^+^ T细胞浸润程度，其最后结果都提示*EGFR*突变型患者肿瘤浸润的CD8^+^ T细胞程度较低。这证明了该结论的可靠性。CD8^+^ T细胞的数量与多种肿瘤患者的预后密切相关，并且，如果这些细胞在肿瘤里的浸润水平低于2.2%，那么患者在手术后出现疾病进展的风险要高出4倍^[7]^。因此较低的CD8^+^ T细胞浸润也可能是*EGFR*突变型肺腺癌患者接受ICIs治疗时疗效差的潜在原因。

同时我们发现*EGFR*突变型患者肿瘤的自然杀伤T细胞浸润程度较高（图 2A），并且较高的自然杀伤T细胞与较好的临床预后存在联系（图 5B）。然后在基因集富集分析的结果中，我们发现了三条与自然杀伤细胞相关的通路在*EGFR*突变患者出现了下调现象。包括自然杀伤细胞介导的对肿瘤细胞的免疫应答的正调控、自然杀伤细胞激活参与免疫反应、在自然杀伤细胞介导的对肿瘤细胞的免疫应答这三条在自然杀伤细胞发挥作用时起到关键作用的通路都下调。同时*EGFR*突变患者记忆B细胞、造血干细胞在肿瘤中浸润的程度较低（图 2A），而较低的记忆B细胞、造血干细胞浸润意味着较差的预后（图 5）。*EGFR*突变患者还具有较高的CD4^+^辅助T细胞2（Th2 T细胞浸润）（图 2A），然而高浸润的Th2 T细胞往往意味着较差的预后（图 5C）。Th2细胞主要分泌白介素4（interleukin-4, IL-4）、IL-5、IL-10、IL-13等，它主导体液免疫应答，辅助抗体生成^[14]^。近来研究^[15, 16]^发现肿瘤组织多分泌Th2类细胞因子，并认为机体处于Th2细胞因子优势状态是肿瘤免疫逃逸的机理之一。而记忆B细胞与造血干细胞浸润在肿瘤免疫反应中的作用还不明确。但这些细胞的浸润程度与肺腺癌患者预后较好存在相关性，因此这两种细胞的缺乏也可能是*EGFR*突变患者对ICIs治疗不敏感的潜在原因。

综上所述，本研究利用TCGA公共数据库，对*EGFR*突变型以及野生型患者的免疫微环境的差异进行了分析，初步探索*EGFR*突变型患者对ICIs治疗获益较差的机制。我们发现*EGFR*突变型患者肿瘤中CD8^+^ T细胞浸润较低，CD8^+^ T细胞作为ICIs的直接效应细胞，其浸润过低可能是导致*EGFR*突变型患者免疫治疗疗效差的直接原因。同时*EGFR*突变型患者肿瘤中还存在记忆B细胞与造血干细胞低浸润，Th2 T细胞高浸润。同时在*EGFR*突变型患者肿瘤中高浸润的自然杀伤细胞出现了功能失调。
